# Complete Genome Sequence of Lactobacillus plantarum subsp. plantarum Strain LB1-2, Isolated from the Hindgut of European Honeybees, Apis mellifera L., from the Philippines

**DOI:** 10.1128/genomeA.00209-18

**Published:** 2018-04-05

**Authors:** M. Fatima C. Ilagan-Cruzada, Albert Remus R. Rosana, Andrew D. Montecillo, Noel G. Sabino, Ida F. Dalmacio

**Affiliations:** a Research Center, Cavite State University, Indang, Cavite, Philippines; b Microbiology Division, Institute of Biological Sciences, University of the Philippines Los Baños, Los Baños, Laguna, Philippines; c Department of Chemistry, University of Alberta, Edmonton, Alberta, Canada; d Departament de Produccio Vegetal I Ciencia Forestal, Universitat de Lleida, Lleida, Spain

## Abstract

Lactobacillus plantarum subsp. plantarum strain LB1-2, isolated from the hindgut of European honeybees in the Philippines, is active against Paenibacillus larvae and has broad activity against several Gram-positive and Gram-negative bacteria. The complete genome sequence reported herein contains gene clusters for multiple bacteriocins and extensive gene inventories for carbohydrate metabolism.

## GENOME ANNOUNCEMENT

Lactobacillus spp. belong to a group of lactic acid bacteria whose members have a low GC content, are Gram positive, and are facultative anaerobic to microaerophilic. This group elicits beneficial effects toward gastrointestinal health and has long been considered a gold standard in probiotic preparations (1). The lactobacilli genomes have shown a high degree of plasticity (2), providing the group competitive advantages in colonizing a wide range of ecological environments, such as in humans, plants, and animals, including honeybees (3). Lactobacillus plantarum subsp. plantarum LB1-2 was isolated form the hindgut of European honeybees, Apis mellifera L., and found to inhibit the growth of Paenibacillus larvae, the causative agent of American foul brood disease in honeybees (4). The 16S rRNA sequence was found to be 99.0% identical to that of L. plantarum LP11F (4), which was isolated from pig gut (5).

Genomic DNA of LB1-2 was extracted and sequenced at Macrogen, Inc. (Seoul, Republic of Korea). Sequencing libraries from the genomic DNA extracts were prepared using the SMRT Cell 8Pac version 3.0 and the DNA polymerase binding kit P6 and sequenced using PacBio RS II technology (Pacific BioSciences, USA). The PacBio reads (1,255,116,911 bp; 370,050 reads) were *de novo* assembled into contigs using the Hierarchical Genome Assembly Process version 3.0 (HGAP3), and the ends of each contig were overlapped to the final genome, which comprised 3,541,869 bp with a GC content of 44.14% and an average sequencing depth of 260×. The assembled genome yielded four replicons composed of one chromosome (3,359 kbp) and three plasmids (117.3, 56.9, and 8.2 kbp). A total of 3,400 predicted coding DNA sequences, 16 rRNAs, and 77 tRNAs were annotated using the NCBI Prokaryotic Genome Annotation Pipeline (PGAP) (https://www.ncbi.nlm.nih.gov/genome/annotation_prok). Species identity was established by calculating the average nucleotide identity (ANI) and *in silico* digital DNA-DNA hybridization (dDDH) using the ANI Calculator (6) and the Genome-to-Genome Distance Calculator version 2.1 (7), respectively, against previously sequenced genomes in the GenBank database. Secondary metabolites were predicted using antiSMASH version 4 (8), while bacteriocins was predicted using BActeriocin GEnome mining tooL version 3 (BAGEL3) (9).

The genome of LB1-2 has ANI and dDDH values of 99.33% and 96.99% (75.51% formula 2), respectively, with L. plantarum subsp. plantarum ATCC 14917^T^, which supports the systematic placement of LB1-2 within this species. The LB1-2 genome encodes gene clusters for the biosynthesis of fusaricidin, terpene, and exopolysaccharides. The genome revealed the presence of gene clusters homologous to multiple bacteriocins, such as plantaricins A, EF, and JK. The genome also revealed a repertoire of genes encoding sugar transport and utilization. The plasmids pLB1-2A, pLB1-2B, and pLB1-2C encode a type I restriction modification system, a type IV secretion system, and hypothetical proteins, respectively. These findings suggest that these gene inventories could play an important part in the interaction of L. plantarum subsp. plantarum LB1-2 with its insect host, the nectar substrate, and bee pathogens. These findings highlight the potential use of L. plantarum subsp. plantarum LB1-2 as a honeybee probiotic.

### Accession number(s).

This whole-genome shotgun project has been deposited in DDBJ/ENA/GenBank under the accession numbers CP025991 for the chromosome and CP025992, CP025993, and CP025994 for the plasmids pLB1-2A, pLB1-2B, and pLB1-2C, respectively.
